# Cervical transcutaneous vagal nerve stimulation (ctVNS) improves human cognitive performance under sleep deprivation stress

**DOI:** 10.1038/s42003-021-02145-7

**Published:** 2021-06-10

**Authors:** Lindsey K. McIntire, R. Andy McKinley, Chuck Goodyear, John P. McIntire, Rebecca D. Brown

**Affiliations:** 1 Infoscitex, Inc., Dayton, OH USA; 2 Air Force Research Laboratory/Applied Neuroscience Branch, WPAFB, OH USA; 3 Air Force Research Laboratory/Security & Intelligence Branch, WPAFB, OH USA

**Keywords:** Biotechnology, Cognitive neuroscience

## Abstract

Fatigue is a pervasive public health and safety issue. Common fatigue countermeasures include caffeine or other chemical stimulants. These can be effective in limited circumstances but other non-pharmacological fatigue countermeasures such as non-invasive electrical neuromodulation have shown promise. It is reasonable to suspect that other types of non-invasive neuromodulation may be similarly effective or perhaps even superior. The objective of this research was to evaluate the efficacy of cervical transcutaneous vagal nerve stimulation (ctVNS) to mitigate the negative effects of fatigue on cognition and mood. Two groups (active or sham stimulation) of twenty participants in each group completed 34 h of sustained wakefulness. The ctVNS group performed significantly better on arousal, multi-tasking, and reported significantly lower fatigue ratings compared to sham for the duration of the study. CtVNS could be a powerful fatigue countermeasure tool that is easy to administer, long-lasting, and has fewer side-effects compared to common pharmacological interventions.

## Abstract (web summary)

Lindsey McIntire et al. assess the impact of noninvasive cervical transcutaneous vagal nerve stimulation (ctVNS) on cognitive performance, mood, and attention in a cohort of sleep-deprived participants. Their results suggest that ctVNS can significantly improve arousal and multi-tasking, and may represent an alternative treatment for fatigue in humans.

## Introduction

Fatigue is a serious and unavoidable problem for many professions such as medicine, transportation, and the military. In general, it is a serious public health risk. Fatigue induced by sustained wakefulness can cause slower reaction times, a reduced ability to multi task, and increases in lapses of attention that can lead to costly, even deadly mistakes^1^. A review by Krueger^2^ found that studies on the behavioral implications of sleep deprivation in humans repeatedly showed increased reaction times, decreased accuracy, decreased attention, and negative alterations in mood. Although some fatigue countermeasures do exist, primarily of a pharmacological nature, these vary in their effectiveness and range of negative side effects, as well as issues of loss of effectiveness with repeated use; therefore, it is important to investigate alternative potential nonpharmacological fatigue countermeasures that can sustain performance longer, with minimal side effects.

One possible way to enhance alertness could be by (non invasively) stimulating an area of the brain called the locus coeruleus (LC). The LC region, located in the brainstem, innervates the entire central nervous system and is the primary source of norepinephrine for the neocortex^3,4^. Referred to as the locus coeruleus–norepinephrine (LC–NE) system, it is believed to play an important role in the regulation of attention, arousal, wakefulness, memory formation, and memory retention^3,5^ many of the behaviors impacted by sleep deprivation. The LC is also recognized as a major wakefulness-promoting nucleus; the activity of the LC positively correlates with the level of arousal^6^. Furthermore, according to Adaptive gain theory, activation of this LC–NE system enhances tonic and phasic arousal^7^. Thus, finding a way to activate this system via nonpharmacological measures could provide a powerful fatigue countermeasure.

Research in this field over the last several years suggests at least one promising potential method: a noninvasive electrical neuromodulation device called transcranial direct current stimulation (tDCS). This technology has been tested for enhancing cognition and human performance under various conditions of lengthy sleep deprivation as well as task-induced fatigue. For example, tDCS has been repeatedly shown to improve performance on a sustained attention task for up to 6 h, improve mood (e.g., fatigue, drowsiness, and vigor), and enhancements of arousal lasting up to 24-h post stimulation from a single 30-min dose of stimulation^8,9^. In addition, tDCS has recently been shown to produce increased activity in the LC region of the brain^10^. Further, various types of attention and arousal behaviors have been shown to be improved or otherwise enhanced by the application of tDCS when compared to control conditions^8,9,11^.

Although tDCS’s effectiveness is suspected to be due to its stimulation of the LC region, this technique may not provide the most direct route or the most effective method for modulating activity in the LC. In its current form, the electric current must first pass through the skin, muscle, skull (which is a large insulator), and cerebrospinal fluid before even reaching the brain. Then, the electricity must travel a lengthy distance through the cortical and subcortical tissues, until it reaches the LC within the brainstem. From the perspective of neuroanatomy, this is quite a lengthy journey. Alternatively, noninvasive stimulation of peripheral nerves that lie just under the surface of the skin and are highly afferent to the LC may offer an easier and more direct path of modulation via electrical stimulation.

One peripheral nerve with afferent connections to the LC is the vagal nerve. Vagal nerve stimulation (VNS) has been an FDA-approved medical treatment for epilepsy and depression for over two decades^12^. More recently, VNS has been shown to improve cognition in animal and human populations. More specifically, VNS stimulation has been shown to significantly improve memory and performance of cognitive tasks in both rats and humans^13–16^. Human subjects receiving invasive VNS for treatment of seizures have shown specific enhancements in memory consolidation leading to better retention^17^. VNS has also been shown to increase neuronal plasticity in humans^18,19^ and leads to increases in the firing rates of noradrenergic neurons in the LC in a rodent population^20^. In small animal models, VNS has been shown to enhance decision-making compared to animals receiving sham stimulation^21^. Most recently, VNS has been shown to accelerate learning for visual target recognition tasks in air force personnel, while simultaneously increasing their attention and arousal^22^ (McKinley, R. A. - manuscript in preparation).

In this study, we delivered cervical transcutaneous vagal nerve stimulation (ctVNS) via a handheld neurostimulation device originally approved to treat cluster headaches and migraines. This device passes a noninvasive electrical current pulsed at 25 Hz through the skin to the nerve via two electrodes placed over the neck. Unlike tDCS, which takes 30 min to dose and is difficult to self-administer, ctVNS is self-administered and only takes 6 min per stimulation dose. Hence, ctVNS potentially provides a quicker, simpler method of stimulating the LC to increase wakefulness, attention,and arousal and improve mood during periods of sleep-deprivation-induced stress.

Most of the research with noninvasive VNS delivers stimulation to the vagus nerve via the auricular branch of the nerve. Because of this, it is largely unknown if ctVNS will produce similar behavioral effects to tDCS by activating the LC–NE pathway. Neurophysiology studies using these noninvasive auricular transcutaneous vagal nerve stimulation (atVNS) devices have attempted to test Adaptive Gain Theory, but these studies have had mixed results. For example, Fischer et al.^23^ found behavioral and N2 event-related potential (ERP) enhancements for atVNS when compared to sham, but did not find a difference in the P300 component (a commonly associated signal with cognition and decision-making). Warren et al.^24^ also found no effect of atVNS on the P300 ERP or on pupil size but found an increase in salivary alpha amylase (sAA) suggesting that atVNS modulates hormonal markers but not psychophysiological indices of the LC–NE pathway. Another study found an effect of atVNS on P300 response and sAA, but the effect was dependent on the target type of the behavioral test^25^. More reliably, functional magnetic resonance imaging (fMRI) studies with atVNS have shown that this type of stimulation adequately activates the vagal pathway and produces greater activation in the brainstem, including at the LC^26–28^. Based on this evidence, we hypothesize that ctVNS will similarly activate this LC–NE pathway, leading to increased arousal during 36 h of sustained wakefulness.

As hypothesized, ctVNS improved some aspects of cognition during the sustained wakefulness vigil. A major finding was that ctVNS significantly improved objective arousal and multitasking for as long as 24-h post-stimulation. Not only did behavioral performance metrics improve but so did subjective ratings of fatigue. This is the first study to successfully utilize ctVNS in healthy humans to enhance cognitive performance during sustained wakefulness. Results and discussion of these findings are detailed below.

## Results

**Fig. 1:**
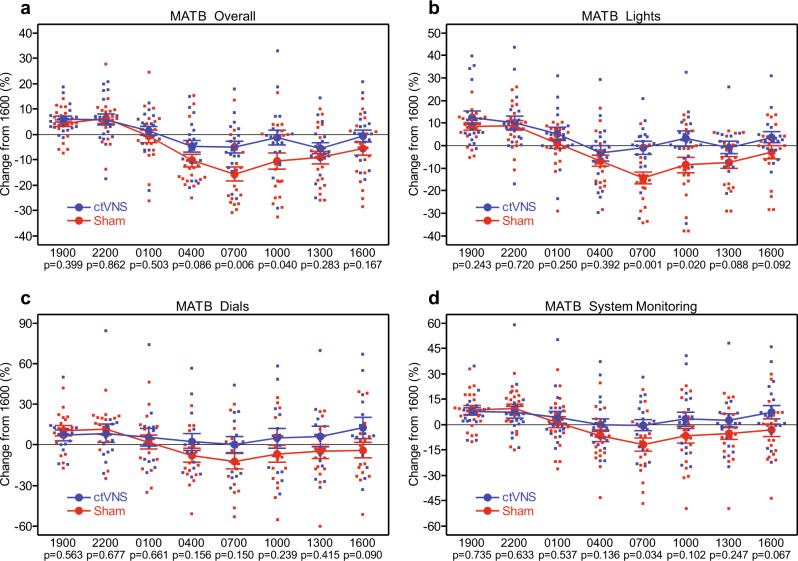
AF–MATB task overall, lights, dials, and system monitoring throughput capacity as a change from 1600. **a** Throughput capacity on all subtasks of the AF–MATB as a change from 1600. **b** Throughput capacity on the lights subtask of the AF–MATB as a change from 1600. **c** Throughput capacity on the dials subtask of the AF–MATB as a change from 1600. **d** Throughput capacity on the system monitoring subtask of the AF–MATB as a change from 1600. Error bars on a–d are mean + /− SE.

A significant interaction of “Group” and “Session” for the AF–MATB task metric of overall throughput capacity was found (Fig. 1, Supplementary Table 1, and Supplementary Data 1). The two-sample *t*-tests revealed a significant difference between the throughput capacity of the ctVNS group and the sham group at 0700 and at 1000 (Fig. 1, Supplementary Table 2, and Supplementary Data 1). The subtask metrics on the AF-MATB are lights, dials, system monitoring, communication, targeting, and resource management. The main effect of “group” was revealed for the “lights” subtask (Fig. 1, Supplementary Table 1, and Supplementary Data 1). The results indicated that the ctVNS group performed significantly better than the sham group. A significant interaction of “group” and “session” on the lights, dials, and system monitoring was found (Fig. 1, Supplementary Table 1, and Supplementary Data 1). The *t*-tests showed that for the lights subtask there was a significant difference between the ctVNS and sham group at 0700 (Fig. 1, Supplementary Table 2, and Supplementary Data 1). The other task metrics were not significant for group or group interaction as shown in Table 1 below.

**Table 1:** Testing schedule for each participant and procedures within each session.

Time, standard and (military)	Activity	Task order within each session (time to complete)
3:30 PM (1530)	Participant arrives	
4:00–5:30 PM (1600–1730)	**Session 1**	**Behavioral tasks:** Mackworth (30 min) N-Back (10 min) PVT (10 min) AF-MATB (20 min) **Subjective questionnaires:** Mood (1 min)
5:30 PM (1730)	Break	
7:00–8:30 PM (1900–2030)	**Session 2 ctVNS administered**	
8:30 PM (2030)	Break	
10:00–11:30 PM (2200–2330)	**Session 3**	
11:30 PM (2330)	Break	
1:00–2:30 AM (0100–0230)	**Session 4**	
2:30 AM (0230)	Break	
4:00–5:30 AM (0400–0530)	**Session 5**	
5:30 AM (0530)	Break	
7:00–8:30 AM (0700–0830)	**Session 6**	
8:30 AM (0830)	Break	
10:00–11:30 AM (1000–1130)	**Session 7**	
11:30 AM (1130)	Break	
1:00–2:30 PM (1300–1430)	**Session 8**	
2:30 PM (1430)	Break	
4:00–5:30 PM (1600–1730)	**Session 9**	
5:30 PM (1730)	Debrief	

**Fig. 2:**
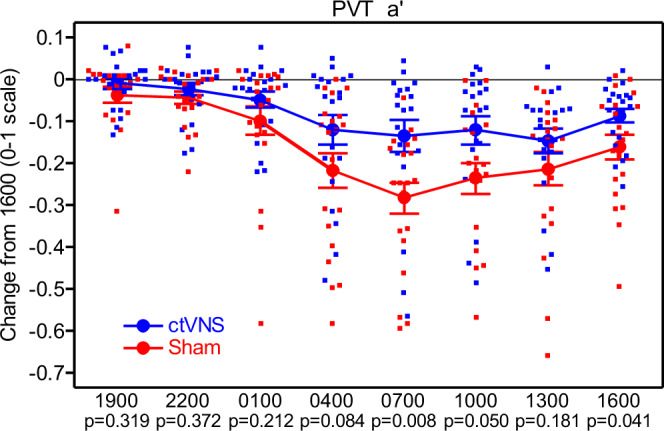
PVT task a’ metric as a change from 1600. PVT *a* prime metric as a change from 1600 with error bars that are mean + /− SE.

A significant main effect of “group” for the PVT task *a’* (*a’* = Hits/(Hits + False Alarms + Lapses)) metric [*F*(1,38) = 5.53, *p* = 0.024] was discovered (see Fig. 2, Supplementary Data 1). This shows that the ctVNS group performed significantly better on the PVT task, for the duration of the study, compared to the sham group. In addition, a significant interaction of “group” and “session” for the PVT task *a’* metric [*F*(7,264) = 2.07, *p* = 0.047] was also found. The *t*-tests did not reveal a significant difference between the performance of the groups at any time point using an adjusted alpha error (see Supplementary Table 3).

There were no significant effects found for the Mackworth Clock Test or N-Back task.

**Fig. 3:**
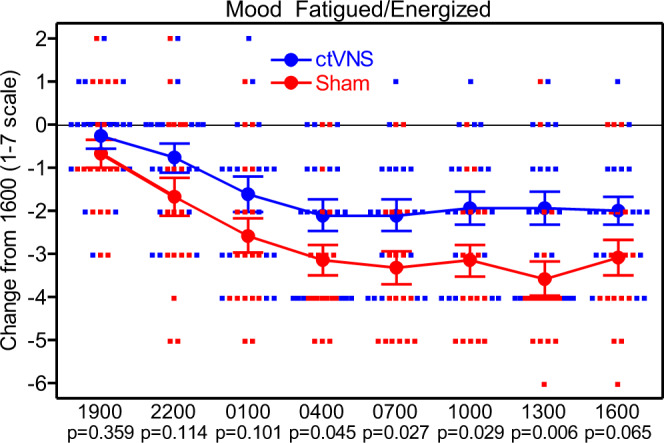
Subjective mood rating of Fatigued/Energized scale as a change from 1600. Subjective mood rating of fatigued versus energized as a change from 1600 with error bars that are mean + /− SE.

A significant main effect of “group” for the Mood questionnaire scale of Fatigued/Energized [*F*(1,35) = 5.44, *p* = 0.026] was found (see Fig. 3, Supplementary Data 1). Participants receiving ctVNS reported lower fatigue ratings and higher energy compared to the sham group. The *t*-tests showed a significant difference between the ratings of fatigued/energized for the ctVNS group and the sham group at 1300 [*t*(35 = 2.92, *p* = 0.006, *d* = 0.99] (see Supplementary Table 4). None of the other scales on this questionnaire were significant.

## Discussion

Many years of research from our own lab has consistently shown that a single 30-min dose of tDCS can sustain various aspects of cognitive performance for as much as 24 h post stimulation during sleep deprivation testing^8,9^. Recent research has shown that the mental performance-enhancing effects in healthy humans may be due in part to activation of the LC brain region^10^. While tDCS remains an effective nonpharmacological option to mitigate the performance decrements associated with sleep loss, there may be alternative noninvasive neuromodulation technologies that target peripheral nerves with the ability to activate the LC more effectively. In addition, peripheral nerve stimulation techniques require less time to administer, the devices are easier for participants to understand and use, and the technique produces very few side effects. The vagus nerve is a peripheral nerve with projections in the ear and neck that have afferent connections to the LC, suggesting other possible sites for noninvasive stimulation in future related work.

The purpose of this study was to investigate the use of peripheral nerve stimulation of the cervical vagus as a fatigue countermeasure. One of the primary findings from this study was a significant interaction for the multitasking test with groups having similar changes in the earlier sessions and significantly different changes later in testing at the 0700 session. Participants had been awake for 24 h during this testing session, which is considered a traditional low point for circadian rhythms; further analysis showed that the group receiving ctVNS stimulation had a significantly higher throughput capacity than the sham group. In fact, the ctVNS group’s throughput capacity was only down 5% from their baseline at 1600 (when they first arrived at the lab, 9-h awake), whereas the sham group’s capacity fell 15%. Twelve total minutes of noninvasive stimulation delivered at 1900 h appears to be providing a long-lasting benefit to multitasking performance, 12 h post stimulation, around the times when performance should theoretically be at its worst.

To our knowledge, the present study is the first to assess multitasking cognitive performance during VNS, and the first to do so under conditions of lengthy sleep deprivation. However, there are other studies in the literature that have tested noninvasive electrical stimulation during multitasking, for instance, tDCS. Using anodal tDCS to enhance multitasking ability, recent studies have shown improvements over sham stimulation^29,30^ but others seem to be suggesting that these results are subtask and location-dependent^31,32^. Both Scheldrup et al.^32^ and Nelson et al.^29^ have results similar to the present study, in that the subtasks requiring visual attention (system monitoring, lights, and dials metrics) seem to be disproportionately affected by stimulation when compared to tasks requiring motor control (targeting metric/visual–motor tracking task) or auditory attention (communication metric/auditory communications monitoring task). Also, the present results specifically show the performance benefits of stimulation peaking at about 12 h post stimulation. Hsu et al.^30^ also found a delayed peak benefit when using tDCS, but the delay was of a matter of minutes as opposed to hours after stimulation. Furthermore, our lab previously examined the effects of tDCS on the same multi-tasking test used in this study, the AF–MATB, and in that previous study, we found no benefit from tDCS during sleep-deprivation testing when compared to sham^33^, while in the present study, we did find a multitasking benefit from ctVNS. This result may suggest that ctVNS provides a more profound benefit in regard to fatigue mitigation than tDCS, at least from a cognitive multitasking perspective.

Given the results from the AF–MATB task that indicates the greatest performance benefit is found for tasks requiring visual attention, it is not surprising that a performance benefit for the visuallybased psychomotor vigilance task (PVT) was also detected. The PVT is a simple reaction time test that requires visual attention, rapid detection, and response, and more generally, measures physiological arousal levels. The results indicate that the ctVNS group performed significantly better than sham on the PVT for the duration of the testing, which by the end of the study, was 19 h after stimulation. In our previous research with tDCS and sleep deprivation, we repeatedly found that tDCS enhanced arousal levels (as measured by the PVT) compared to caffeine and sham stimulation for as long as 24 h post stimulation^8,9^. Furthermore, when we delivered cervical tVNS on 4 consecutive days using the same device and the same stimulation procedures from this study, we found elevated PVT accuracy scores as long as 90 days post stimulation^22^.

There is a large body of research surrounding the impact of fatigue on cognition in healthy individuals. Some research focuses on specific types of cognition that degrade with increasing levels of fatigue. For example, it is well known that multitasking ability, reaction time, and accuracy decrease with increasing levels of fatigue^1^. It is also known that activation of the LC–NE pathway enhances arousal, vigilance, and attention^3,5^. Given our positive results on various behavioral and cognitive tests related to arousal, vigilance, and attention, as measured by multitasking, reaction times, and accuracy, it is reasonable to suspect that the present method of stimulating the cervical transcutaneous vagal nerve is indeed activating the LC–NE pathway, as hypothesized. However, future studies are recommended to convincingly confirm this suspected link with different lines of evidence (e.g., neurophysiological).

A complementary body of research suggests that fatigue impacts performance not on all cognitive tasks in a general sense, but more specifically on tasks that are not well-learned, not executed automatically, require deliberate action and thought, and are more mentally demanding^34^. This research appears to suggest that the cognitive performance decrements as a result of increasing fatigue are due to a deterioration of executive control^34^. Executive control is “the ability to regulate perceptual and motor processes in order to respond in an adaptive way to novel or changing task demands”^35^. In other words, executive control is more commonly associated with tasks that require effortful attention, decision-making, and complex cognitive abilities like memory, planning, and mental flexibility. Our results support this conceptualization of fatigue’s effects on the mind, in that the tasks that were most susceptible to fatigue also require the most executive-control type of cognitive resources to accomplish. For example, the AF–MATB task in general requires constant vigilance and effortful attention divided across multiple subtasks with more complex actions beyond simple stimulus-response button pressing such as; rapid decision-making, multi button responses interspersed throughout the keyboard, and manual analog control stick inputs all intermixed over time. The subtasks that required the most vigilance, rapid decision-making, and cognitive resources like memory, planning, and mental resource allocation to track changing complex information over time were the lights, dials, and system monitoring subtasks. Our results showed that these were the subtasks most susceptible to degraded performance associated with increasing levels of fatigue. Thus, we interpret these data overall to suggest that an intervention to enhance cognitive arousal like ctVNS was able to raise performance back to baseline levels in these subtasks, compared to sham intervention, by advantageously stimulating the executive control system.

**Fig. 4:**
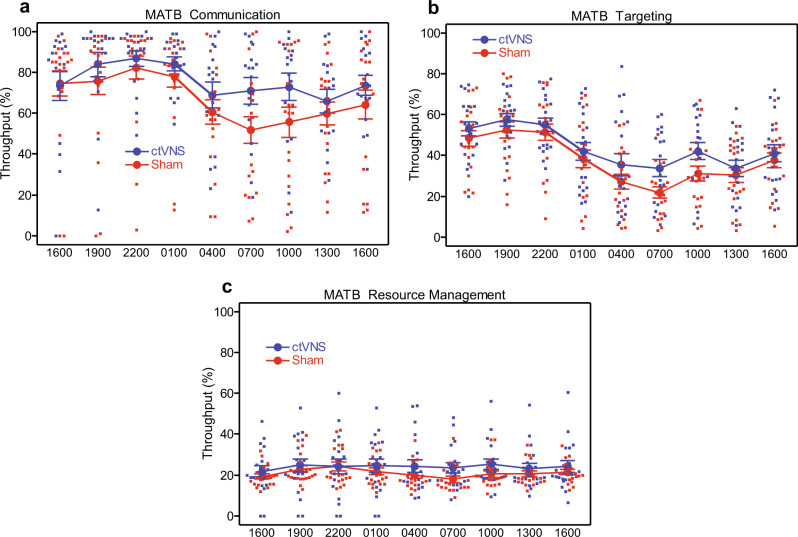
AF-MATB subtasks: communication, targeting, and resource management. **a** AF-MATB communication subtask throughput capacity percentage. **b** AF-MATB targeting subtask throughput capacity percentage. **c** AF-MATB resource management subtask throughput capacity percentage. For graphs a–c, error bars are mean +/– SE.

The other subtasks on the AF–MATB (communication, targeting, and resource management) are not as distracting (communication was an audio-based listening task, while all other tasks were visual-based tasks), not as cognitive (targeting was a visually guided manual control task and could be automatically executed by the motor control system by dissociating from executive function), or not as difficult (resource management is considered on the AF-MATB to be the “simplest” task and requires less constant vigilance). For further consideration, take the example of the communication subtask. This is an auditory task in which the participant listens for a sporadically occurring verbal command, then simply follows the command by manually entering the requested information into the subtask window. In this case, the auditory signals interfere less with the visual channel, allowing other tasks to be accomplished in parallel. Also, the task requires use of only a small chunk of short-term auditory/verbal memory, for a brief period, until the participant manually enters the requested code into the prompt (using up/down arrow buttons to select one of several possible codes). Once that is accomplished, that channel requires no further monitoring until the participant receives another verbal command. There is simply not much in the way of complex cognition or executive control required to accomplish this subtask and little distraction for the other visually-based subtasks. Therefore, the tasks less susceptible to performance decrements as a result of sleep deprivation donot appear to benefit from a countermeasure like ctVNS since the performance “boost” back to normal baseline levels is not needed (see Fig. 4, Supplementary Data 1).

Researchers have previously found an effect of VNS on working memory and attention^13,15^; however, in this study, we did not find an effect of ctVNS on working memory/attention. This lack of effect could be due to differences in population (clinical versus nonclinical; or perhaps non-military versus military), differences in the tasks used to asses these behaviors, or differences in the type of VNS utilized (invasive/transcutaneous, cervical/auricular, or stimulation parameter differences). Future research should explore these differences further in healthy populations and perhaps without sleep deprivation stress.

In a previous study, we utilized the same stimulation device and the same attention task as was used in this experiment, but in that study, participants were not sleep-deprived and we found no effect of cervical tVNS on attention during the 4 days of consecutive stimulation^22^. Furthermore, a review of the effects of sleep deprivation on working memory has found conflicting results regarding their association^36^. Their review found that decreased performance on working memory tasks is associated with reductions in neural activation; however, they also discussed evidence for a compensatory mechanism that can cause increased activation under sleep deprivation stress that allows for performance levels to be maintained, despite decreases inactivation. Unfortunately, the present experiment does not shed any further clarifying light on this issue.

Another interesting finding from this study was the smaller increase in subjective fatigue rating by participants who received ctVNS when compared to sham. Other researchers have found in clinical populations that the VNS improved mood^37,38^. In tDCS studies, too, our lab has discovered improvements in subjective mood and decreased subjective fatigue during sleep deprivation stress^8,22^. Given the local proximity of the ophthalmic (V1, sensory) branch of the trigeminal nerve to the anode used in our tDCS paradigms, it is reasonable to suggest that perhaps some of the current may have innervated this peripheral nerve. Because the trigeminal nerve also has direct projections to the LC, it is possible that tDCS and peripheral nerve stimulation may share the same stimulation pathway to the LC. In fact, a recent study by Asamoah, Khatoun, and McLaughlin^39^ found that the primary effects of transcranial alternating current stimulation (tACS) are caused by innervation of peripheral nerves rather than current flowing into the brain directly.

In practice, cervical tVNS devices are easier to use due to the fact that the electrodes do not need to be placed over hair and produce a much shorter stimulation session. This reduces difficulties in achieving and maintaining low impedance during the stimulation session. In addition, peripheral nerve stimulation techniques may provide a more “direct” method of activating the LC, which reduces the possibility of stimulating other nontargeted brain regions. Future research with ctVNS should investigate other forms of cognitive enhancements than those measured here, and consider biomarker or imaging analysis to more deeply explore the mechanisms of action, determine why low-current neural stimulation seems to be effective, and what neural sites and regions are being differentially activated. A study that directly compares ctVNS to other forms of noninvasive electrical stimulation and its effects on cognition, performance, and mood should be considered.

At this time, it remains unclear whether noninvasive auricular vagal nerve stimulation produces similar behavioral effect as cervical tVNS. Future studies should examine the behavioral effects of stimulation across the different branches of the vagus. A limitation of this study is the sample population; this research was conducted exclusively on the active-duty military who tend to be younger, healthier, and predominately male compared to the general population. Another limitation of this current study is the inability to directly compare it to other similar research since few experimental studies on this device have been conducted thus far. In fact, cervical tVNS is perhaps the least common form of VNS researched in the literature, and the device we utilized for testing is currently exclusively researched in only clinical populations, apart from our own lab’s experimental work in healthy normal subjects. Therefore, little is known about the appropriateness of the sham, the dosage, and how well this technology would transfer to more real-world settings. Future research should focus on dosage (time-of-day, duration, frequency, strength, etc.), sham techniques (off-target stimulation, pulse at a different frequency, etc.), and expanding the population and tasks into more realistic settings. Other researchers may also want to consider verifying that this ctVNS device is indeed activating the LC–NE pathway, as suspected, by collecting neurophysiological or biological indices such as ERP, sAA, or fMRI data.

## Methods

### Equipment

#### ctVNS stimulator

**Fig. 5:**
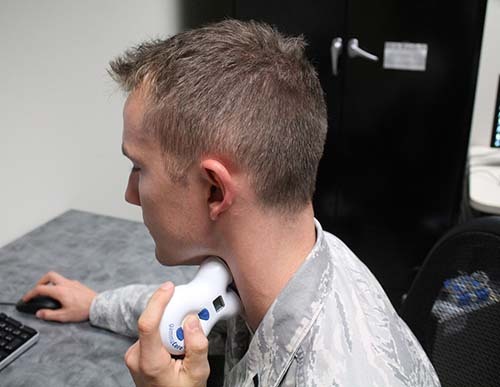
gammaCore® Device-use demonstration. An airmen demonstrating the use of the gammaCore® ctVNS device.

The ctVNS device used in this study is the gammaCore® product that is FDA-approved to treat cluster headaches and treatment-resistant migraines. This device passes a noninvasive electrical current pulsed at 25 Hz through the skin to the nerve via two electrodes placed over the neck. It is a commercially available electrical vagal nerve stimulation device developed by electroCore®, Inc. (Rockaway, NJ, USA, see Fig. 5). This noninvasive device provides an electrical current pulsed at 25 Hz and automatically shuts off after 2 min of stimulation. Sham stimulation was delivered via a sham device that looked identical to an active device and was provided by the same manufacturer. The sham device provided a similar clicking sound and tactile vibrations as the active device but did not deliver any current. Double blinding was accomplished by ensuring that the researcher directly assisting the participant with data collection did not know which device was being used (a different researcher performed the initial setup for each collection session).

### Subjects

Forty active-duty military participants from Wright-Patterson Air Force Base completed this study. They were randomly assigned to one of two groups (ctVNS stimulation versus sham). There were 33 male and 7 female participants with an average age of 28 ± 6 years. Participants were compensated for their time. Volunteers were screened prior to participation and disqualified if they met any of the exclusion criteria described in McKinley et al.^40^. Examples of the exclusion criteria included any neurological diagnosis, any psychological diagnosis/hospitalization, nonremovable metal around the head, uncorrected vision impairments, sleep disorders, pregnant or trying to become pregnant, smoking, history of frequent headaches and/or migraines, history of seizures, history of fainting,and high blood pressure or heart disease even if controlled with medication and currently taking psychotropic or opioid medications. Forty-two participants passed initial screening and were thus initially enrolled in the study, but one participant withdrew immediately prior to data collection due to time constraints, and another participant started the study but withdrew before completion, so only forty participants’ data were usable in the final analyses. The 20 participants in the “treatment” group received active ctVNS stimulation, while the 20 participants in the control group instead received sham stimulation. Both groups underwent 34 h of continuous sleep deprivation.

### Performance tasks

Participants were required to complete 4 performance tasks and answer subjective questionnaires throughout the duration of this study. The tasks are described below.

#### Mackworth clock test (Sustained attention task)

This vigilance task was developed according to the description of the task used by Kilpelӓinen, Huttunen, Lohi, and Lyytinen^41^. The task was a modified version of the Mackworth clock test with parameters adapted from Teikari^42^ and ran on a standard desktop computer. The participant was presented a visual display with 16 hole-like black circles arranged in a clock-like figure against a black background. Each circle changed from black to red for 0.525 sec in turn, with each cycle lasting 3 sec. The red light moved in a clockwise sequence by one step, which was considered the normal stimulus appearance. When the light moved twice the usual distance (i.e., skipping a circle), it was considered a critical signal, and the participant was required to respond to this signal by pressing the spacebar as fast as possible on the keyboard with his or her preferred index finger. Participants performed this task for 30 min every session. Twelve critical signals occurred at randomized times within each session.

#### N-back task

Participants were required to perform the N-back task during testing to assess working memory activity. This involved asking participants to remember a series of letters presented to them on a computer screen, one by one, and to respond when the current letter was presented N trials earlier (for this study, 2 trials back). A new letter was presented for 200  ms every 3 sec. Participants performed this task for 10 min every session.

#### Psychomotor vigilance task (PVT)

Participants were required to perform the PVT task during testing. The PVT-192 (Ambulatory Monitoring, Inc.; Ardsley, NY) is an 8” × 4/5” × 2/4” handheld, battery-operated computerized test presentation and data capture system that records visual reaction times. The visual stimulus was presented on a small liquid crystal display (LCD) that presented a number counted up by milliseconds. The stimulus was presented for up to 1 min (60,000 msec), allowing the participant to respond by using a button press with the thumb. Once the participant pressed the button, the device recorded the reaction time of the stimulus. The interstimulus interval varied randomly from 2 to12 sec. The task was 10 min in duration. The PVT required sustained attention and discrete motor responses. A “Hit” was defined as a correct response to the stimulus with a reaction time greater than or equal to 150 ms and less than or equal to 500 ms. Any response greater than 500 ms was considered a “Lapse” and any response that was less than 150 ms was classified as a “False Alarm”.

#### Air Force–Multi-Attribute Task Battery (AF-MATB)

This task was originally developed by the National Aeronautics and Space (NASA) to evaluate human performance metrics during a multitasking test paradigm^43^. The AF-MATB is a modified version of the original task created by NASA that incorporates the Human Operator Informatic Model that evaluates both human performance and strategy on multitasking^44–46^. The model calculates the amount of information an operator can process as “throughput capacity” by calculating the differences between the number of stimuli displayed versus the number of stimuli to which the participant responded. The task requires the operator to simultaneously monitor and respond to four separate cognitive process tasks shown on a visual monitor, with each task in a separate quadrant of the display. The cognitive processes tested included a visual system alert monitoring task (lights, dials, and system monitoring metric), a visual–motor tracking task (targeting metric), an auditory communication monitoring task (communication metric), and a visual resource management task (resource management metric). Therefore, there are four subtasks that test four different aspects of cognition and from those four subtasks, we get six different metrics (1 from each subtask, except for the visual system alert monitoring task has 3 metrics). We also computed an overall score metric for the entire task that included the 6 aforementioned metrics. Participants performed the AF-MATB program for 20 min at a medium-high-difficulty level.

### Subjective questionnaire

#### Mood questionnaire

A 15-item mood questionnaire was also administered. The participants checked a box closer to the mood on the scale that they most identified with at the moment. For example, “Fatigued or Energized”, “Happy or Sad”, and “Optimistic and Pessimistic” were items on this questionnaire. Depending on the box selected, the questionnaire would output a numerical score (1–7) to quantify the mood.

### Procedures

Before any study procedures were carried out, the Air Force Research Laboratory institutional review board on human research approved this study and informed consent was obtained from all volunteers. Participants were randomly assigned to one of two groups for this placebo-controlled, double-blinded study. At 1900, Group 1 received active ctVNS on the skin over the left and right cervical vagal nerve (neck) at 25 Hz for 2 min on each side (with a 2-min break in- between). Group 2 received sham ctVNS at 1900 on both the left and right cervical vagal nerve. The sham was conducted with a separate sham ctVNS device provided by the manufacturer. The sham provides similar sensations (e.g., vibrations) without providing electrical stimulation of the nerves.

After consenting, participants filled out a medical screening questionnaire and received training on the tasks. Training consisted of verbal instructions and visual demonstrations of each task. Prior to being dismissed from the training session, the participants were instructed to get at least 7 h of sleep for the night(s) prior to their sleep deprivation testing session. This was verified upon arrival for the testing session by a sleep watch the participants were required to wear while sleeping.

**Fig. 6:**
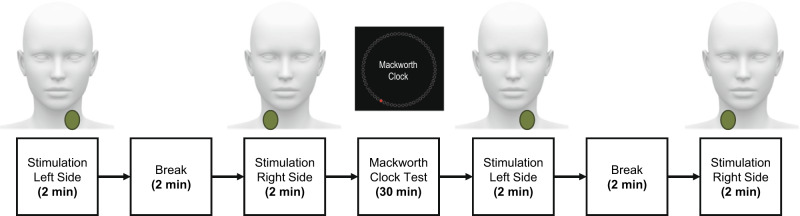
Flow chart of ctVNS delivery during session 2 occurring at 1900 (7:00 PM Local). Illustration of ctVNS stimulation paradigm used during session 2 at 1900 (7:00 PM Local).

On the day of testing, participants were required to wake by 0700 and report to the lab at 1530. They were also instructed to not nap and not to consume any caffeine or central nervous system-altering medications/substances on the experimental testing day. At 1600, after adequate sleep was verified by analyzing the data from the watch, participants performed Session 1 of the behavioral and subjective testing. The order of the tasks was Mackworth Clock test, N-Back, PVT, AF-MATB, and subjective questionnaire. At 1730, participants received a 1.5-h break where they could watch movies, play video games, read, talk, eat, etc. They were not allowed to nap or consume caffeine for the duration of the study. At 1900, Session 2 of the behavioral and subjective testing began. At the start of Session 2, the participants received the ctVNS stimulation on one side of their neck followed by a 2 min break and then 2 min of stimulation on the other side of the neck. Next, they performed the 30-min Mackworth Clock task and then received a second dose of ctVNS following the exact procedures described above (see Fig. 6 flow chart). The remainder of the behavioral and subjective testing was then completed before the next break began (at 2030). Session 3 began at 2200 h and was exactly the same as the first and second sessions with the exception of no stimulation. Stimulation was only delivered during the Session 2 of this study. The procedures were repeated every three hours for a total of 9 data collection tasking sessions. Participants were released at approximately 1730 h on day 2 for a total of 34 h of continuous wakefulness (see Table 1 for detailed procedures). They were required to be driven home by a rested friend or family member.

### Statistics and reproducibility

A two-tailed two-sample *t*-test at the first session (1600 h) determined there were no significant differences (*p* < 0.05) between groups (*n* = 20 for each group) for all variables analyzed, thus indicating baselines were similar for the two groups before the stimulation intervention. A mixed-effects model ANOVA was performed using change from 1600 h as the dependent variable with “Group” (ctVNS, Sham), a between-factor, and “Session” (8 levels from 1900–1600) a within- factor. If the Group-by-Session interaction was significant (*p* < 0.05), a two-tailed two-sample *t*-test was performed at each session comparing groups. A Bonferroni alpha-error adjustment was used to adjust for multiple testing with 8 sessions, resulting in a per-comparison error level of 0.05/8 = 0.0063. In the figures presented below, a single star (*) below the session time represents *p* < 0.0063. Tables 3–5 show *t*-test results for all sessions where *p* < 0.05. Cohen’s *d* is provided to show effect size.

### Reporting summary

Further information on research design is available in the Nature Research Reporting Summary linked to this article.

## Supplementary information

Supplementary Information

Description of Additional Supplementary Files

Supplementary Data 1

Reporting Summary

## Data Availability

Data related to Figs. 1–4 are presented in Supplementary Data 1. Other raw data supporting the conclusions of this paper are property of the United States Government and require clearance through Public Affairs prior to release. Please contact the corresponding author if you wish to obtain the raw data. L.K.M. and R.A.M. will seek public affairs clearance for individuals. If release is granted, data will be made available by the authors, without undue reservation, to any qualified researcher.
