# Author Correction: Cervical transcutaneous vagal nerve stimulation (ctVNS) improves human cognitive performance under sleep deprivation stress

**DOI:** 10.1038/s42003-021-02377-7

**Published:** 2021-07-01

**Authors:** Lindsey K. McIntire, R. Andy McKinley, Chuck Goodyear, John P. McIntire, Rebecca D. Brown

**Affiliations:** 1 Infoscitex, Inc., Dayton, OH USA; 2 Air Force Research Laboratory/Applied Neuroscience Branch, WPAFB, OH USA; 3 Air Force Research Laboratory/Security & Intelligence Branch, WPAFB, OH USA

**Keywords:** Biotechnology, Cognitive neuroscience

Correction to: *Communications Biology* 10.1038/s42003-021-02145-7, published online 10 June 2021.

The original version of this Article contained an error in Fig. 2, in which the *y* axis was titled, “Change from 1600 (%)”. The correct *y* axis title is “Change from 1600 (0–1 scale)”. The correct version of Fig. 2 is:


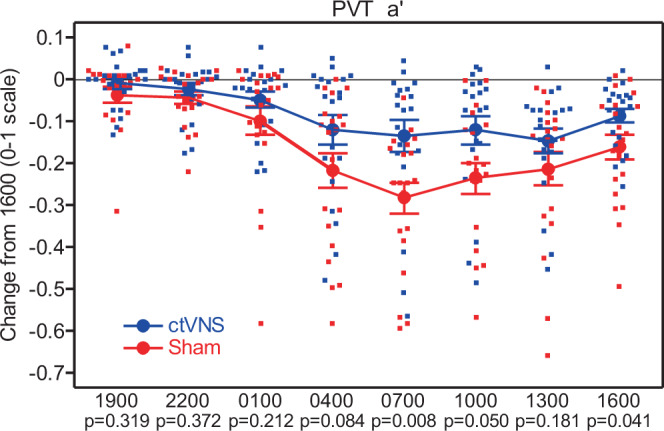


which replaces the previous incorrect version:


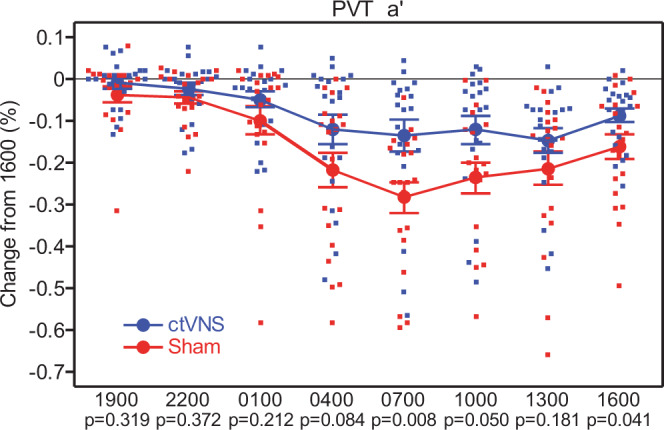


The original version of this Article also contained an error in Fig. 3, in which the *y* axis was titled, “Change from 1600 (%)”. The correct *y* axis title is “Change from 1600 (1–7 scale)”. The correct version of Fig. 3 is:


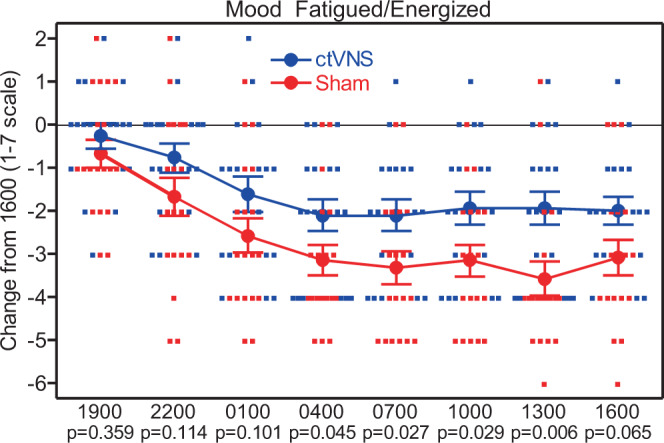


which replaces the previous incorrect version:


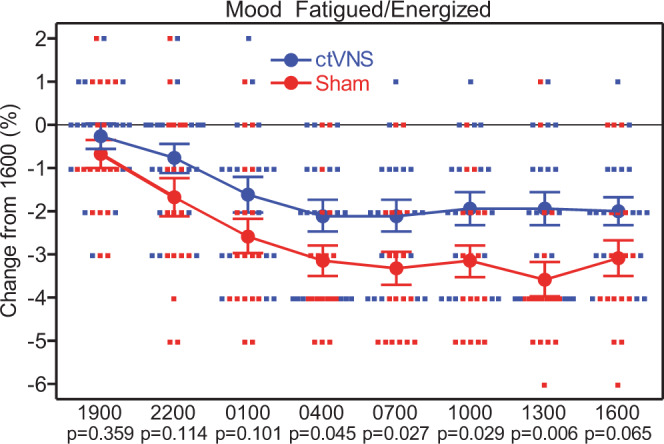


Both errors have now been corrected in the HTML and PDF versions of the Article.

