# Supplementary material for: Cervical transcutaneous vagal nerve stimulation (ctVNS) improves human cognitive performance under sleep deprivation stress
Source: Commun Biol. 2021 Jun 10;4:634. doi: 10.1038/s42003-021-02145-7 (PMC8192899; doi:10.1038/s42003-021-02145-7)
Supplement: Supplementary file 1 — Supplementary Information [file 42003_2021_2145_MOESM1_ESM.pdf]

Supplementary Information for:

Cervical Transcutaneous Vagal Nerve Stimulation (ctVNS) Improves Human Cognitive Performance under Sleep Deprivation Stress

*Lindsey K. McIntire[1], R. Andy McKinley[2], Chuck Goodyear[1], John P. McIntire[3], Rebecca D. Brown[1]

[1]Infoscitex, Inc., 4027 Colonel Glenn Highway, Suite 210, Dayton, OH 45431, USA
lindsey.mcintire.ctr@us.af.mil, charles.goodyear.1@us.af.mil, rebecca.brown.5.ctr@us.af.mil

[2]Air Force Research Laboratory/Applied Neuroscience Branch, 2510 Fifth Street, Building 840, WPAFB, OH 45433, USA, richard.mckinley.2@us.af.mil

[3]Air Force Research Laboratory/Security & Intelligence Branch, 2401 Avionics Circle, Building 620, WPAFB, OH 45433, USA, john.mcintire.1@us.af.mil

*Corresponding Author: Lindsey McIntire, 4027 Colonel Glenn Highway, Suite 210, Dayton, OH 45431, USA, 937-938-3609, lindsey.mcintire.ctr@us.af.mil

Supplementary Table 1. Mixed-Effects Model ANOVA Table, for a change from 1600 with Main Effects and Significant Interactions in Grey for the AF-MATB Task

Dependent Variable	Source	DF	DFe	F	p
Lights	Group	1	37	4.90	0.0331
	Session	7	257	22.41	<.0001
	Group x Session	7	257	2.21	0.0338
Dials	Group	1	29	1.01	0.3233
	Session	7	201	6.73	<.0001
	Group x Session	7	201	2.98	0.0054
System Monitoring	Group	1	37	1.82	0.1860
	Session	7	257	14.12	<.0001
	Group x Session	7	257	3.55	0.0012
Communication	Group	1	34	1.66	0.2068
	Session	7	236	12.57	<.0001
	Group x Session	7	236	1.05	0.3984
Targeting	Group	1	37	0.25	0.6182
	Session	7	257	49.63	<.0001
	Group x Session	7	257	1.69	0.1122
Resource Management	Group	1	36	0.05	0.8207
	Session	7	250	2.40	0.0218
	Group x Session	7	250	1.55	0.1520
Overall	Group	1	37	3.15	0.0843
	Session	7	257	30.73	<.0001
	Group x Session	7	257	3.00	0.0047

Supplementary Table 2. Two-tailed two-sample *t*-test and Cohen's *d* Values for the AF-MATB Task

Dependent Variable (change from 1600)	Session	Sham		ctVNS		Two-sample *t*-test			Cohen's *d*
		Mean	SE	Mean	SE	*t*	DF	*p*	
Lights	0700	-14.4	2.6	-0.9	2.9	3.45	37	0.0014	1.13
	1000	-8.5	3.5	3.1	3.3	2.44	37	0.0195	0.80
System Monitoring	0700	-11.8	3.9	-0.4	3.3	2.20	37	0.0341	0.72
Overall	0700	-15.6	2.7	-5.0	2.4	2.90	37	0.0063	0.95
	1000	-10.5	3.1	-1.2	3.0	2.13	37	0.0397	0.70

Supplementary Table 3. Two-tailed two-sample *t*-test and Cohen's *d* Values for the PVT Task

Dependent Variable (change from 1600)	Session	Sham		ctVNS		Two-sample *t*-test			Cohen's *d*
		Mean	SE	Mean	SE	*t*	DF	*p*	
a'	0700	-0.28	0.04	-0.14	0.04	2.81	38	0.0078	0.91
	1600	-0.16	0.03	-0.09	0.02	2.14	30.4	0.0406	0.70

Supplementary Table 4. Two-tailed two-sample *t*-test and Cohen's *d* Values for Mood

Dependent Variable (change from 1600)	Session	Sham		ctVNS		Two-sample *t*-test			Cohen's *d*
		Mean	SE	Mean	SE	*t*	DF	*p*	
Fatigued/ Energized	0400	-3.16	0.35	-2.11	0.36	2.08	35	0.0454	0.70
	0700	-3.32	0.38	-2.11	0.36	2.31	35	0.0268	0.78
	1000	-3.16	0.36	-1.94	0.39	2.28	35	0.0287	0.77
	1300	-3.58	0.40	-1.94	0.39	2.92	35	0.0061	0.99
