# Supplementary material for: Cervical transcutaneous vagal nerve stimulation (ctVNS) improves human cognitive performance under sleep deprivation stress
Source: Commun Biol. 2021 Jun 10;4:634. doi: 10.1038/s42003-021-02145-7 (PMC8192899; doi:10.1038/s42003-021-02145-7)
Supplement: Supplementary file 2 — Description of Additional Supplementary Files [file 42003_2021_2145_MOESM2_ESM.pdf]

Descriptions of Additional Supplementary Files

Supplementary Data 1
Description: Source Data for figures.
