# Supplementary material for: Cervical transcutaneous vagal nerve stimulation (ctVNS) improves human cognitive performance under sleep deprivation stress
Source: Commun Biol. 2021 Jun 10;4:634. doi: 10.1038/s42003-021-02145-7 (PMC8192899; doi:10.1038/s42003-021-02145-7)
Supplement: Supplementary file 4 — Reporting Summary [file 42003_2021_2145_MOESM4_ESM.pdf]

nature research

Corresponding author(s): Lindsey McIntire

Last updated by author(s): 04/13/2021

Reporting Summary

Nature Research wishes to improve the reproducibility of the work that we publish. This form provides structure for consistency and transparency in reporting. For further information on Nature Research policies, see our Editorial Policies and the Editorial Policy Checklist.

Statistics

For all statistical analyses, confirm that the following items are present in the figure legend, table legend, main text, or Methods section.

n/a	Confirmed	
☐	☒	The exact sample size (*n*) for each experimental group/condition, given as a discrete number and unit of measurement
☐	☒	A statement on whether measurements were taken from distinct samples or whether the same sample was measured repeatedly
☐	☒	The statistical test(s) used AND whether they are one- or two-sided<br>*Only common tests should be described solely by name; describe more complex techniques in the Methods section.*
☐	☒	A description of all covariates tested
☐	☒	A description of any assumptions or corrections, such as tests of normality and adjustment for multiple comparisons
☐	☒	A full description of the statistical parameters including central tendency (e.g. means) or other basic estimates (e.g. regression coefficient) AND variation (e.g. standard deviation) or associated estimates of uncertainty (e.g. confidence intervals)
☐	☒	For null hypothesis testing, the test statistic (e.g. *F*, *t*, *r*) with confidence intervals, effect sizes, degrees of freedom and *P* value noted<br>*Give P values as exact values whenever suitable.*
☒	☐	For Bayesian analysis, information on the choice of priors and Markov chain Monte Carlo settings
☒	☐	For hierarchical and complex designs, identification of the appropriate level for tests and full reporting of outcomes
☐	☒	Estimates of effect sizes (e.g. Cohen's *d*, Pearson's *r*), indicating how they were calculated

Our web collection on statistics for biologists contains articles on many of the points above.

Software and code

Policy information about availability of computer code

Data collection	We utilized pre-programmed tasks that can be commercially purchased or supplied for free depending on the task.
Data analysis	SASS version 9.2

For manuscripts utilizing custom algorithms or software that are central to the research but not yet described in published literature, software must be made available to editors and reviewers. We strongly encourage code deposition in a community repository (e.g. GitHub). See the Nature Research guidelines for submitting code & software for further information.

Data

Policy information about availability of data

All manuscripts must include a data availability statement. This statement should provide the following information, where applicable:

- Accession codes, unique identifiers, or web links for publicly available datasets
- A list of figures that have associated raw data
- A description of any restrictions on data availability

Data from this study is property of the U.S. Government and will be made available if approved for public release by public affairs.

Field-specific reporting

Behavioural & social sciences study design

All studies must disclose on these points even when the disclosure is negative.

Study description	The experimental design for this study was a single factor design tested at two levels (active vs sham), between subjects, and double blinded. The data collected was qualitative as it was cognitive performance tasks and subjective questionnaires to assess mood.
Research sample	Forty male (n=33) and female (n=7) active-duty military participants between the ages of 22-34 participated in this study. This is a representative sample of a typical military population. We are restricted to testing active duty military only by our IRB.
Sampling strategy	Subjects were randomly assigned to sham or active stimulation condition. Sample size was determined by the N-Back task used in a recently completed study involving pre to post performance. This task will be a primary task in the proposed study, also involving pre to post performance. The primary dependent variable from this task is response time. In the completed study, mean pre was 1465 ms and the pooled standard deviation of the pre to post change was 242 ms. Based on previous research, it is desired to detect a difference between 2 groups of 15% in the change from pre to post. Using a two-tailed 2-sample t-test with alpha error=0.05 and power=0.8, 20 subjects per group are required to detect the 15% difference in change from pre to post.
Data collection	Cognitive performance data and subjective mood data were all captured using standard desktop computers. All researchers in the room with the participant were blinded to the condition the participant was assigned to. Subjects completed 9 sessions of data collection while sleep deprived. During their 1-hour breaks between sessions they could talk, watch movies, play video games, etc. They could not have caffeine or any other CNS-altering substance.
Timing	Data collection began in Sept of 2019 and concluded in March 2020. There was no pause or stop to the collection in between these time periods.
Data exclusions	The only data excluded from the analysis was missing data. Missing data occurred rarely due to missing an item on a questionnaire, pressing the wrong button for task response, or a computer glitch not recording the data.
Non-participation	Two participants dropped out of the study. One dropped out prior to data collection due to time constraints in their work schedule. Another dropped out during data collection due to being too tired and not feeling like they could complete.
Randomization	Participants were randomly assigned by a flip of the coin when they arrived for consent/training day.

Reporting for specific materials, systems and methods

We require information from authors about some types of materials, experimental systems and methods used in many studies. Here, indicate whether each material, system or method listed is relevant to your study. If you are not sure if a list item applies to your research, read the appropriate section before selecting a response.

Materials & experimental systems

n/a	Involved in the study	
x		Antibodies
x		Eukaryotic cell lines
x		Palaeontology and archaeology
x		Animals and other organisms
	x	Human research participants
x		Clinical data
x		Dual use research of concern

Methods

n/a	Involved in the study	
x		ChIP-seq
x		Flow cytometry
x		MRI-based neuroimaging

Human research participants

Policy information about studies involving human research participants

Population characteristics	See above
Recruitment	Recruitment was done via email through a mass email distribution system that went to all active-duty military stationed at Wright-Patterson Air Force Base. All who requested to participate were able if spots remained in the study. No one was excluded based on biased recruitment. Only people excluded were those who did not meet the medical screening form for safety and health reasons.
Ethics oversight	Air Force Research Laboratory IRB

Note that full information on the approval of the study protocol must also be provided in the manuscript.
